# Conserved residues in Ycf54 are required for protochlorophyllide formation in *Synechocystis* sp. PCC 6803

**DOI:** 10.1042/BCJ20161002

**Published:** 2017-02-20

**Authors:** Sarah Hollingshead, Sophie Bliss, Patrick J. Baker, C. Neil Hunter

**Affiliations:** Department of Molecular Biology and Biotechnology, University of Sheffield, Firth Court, Western Bank, Sheffield S10 2TN, U.K.

**Keywords:** chlorophyll, Mg-protoporphyrin IX monomethylester cyclase, oxidative cyclase, photosynthesis, synechocystis, Ycf54

## Abstract

Chlorophylls (Chls) are modified tetrapyrrole molecules, essential for photosynthesis. These pigments possess an isocyclic E ring formed by the Mg-protoporphyrin IX monomethylester cyclase (MgPME–cyclase). We assessed the *in vivo* effects of altering seven highly conserved residues within Ycf54, which is required for MgPME–cyclase activity in the cyanobacterium *Synechocystis*. *Synechocystis* strains harbouring the Ycf54 alterations D39A, F40A and R82A were blocked to varying degrees at the MgPME–cyclase step, whereas the A9G mutation reduced Ycf54 levels by ∼75%. Wild-type (WT) levels of the cyclase subunit CycI are present in strains with D39A and F40A, but these strains have lowered cellular Chl and photosystem accumulation. CycI is reduced by ∼50% in A9G and R82A, but A9G has no perturbations in Chl or photosystem accumulation, whilst R82A contains very little Chl and few photosystems. When FLAG tagged and used as bait in pulldown experiments, the three mutants D39A, F40A and R82A were unable to interact with the MgPME–cyclase component CycI, whereas A9G pulled down a similar level of CycI as WT Ycf54. These observations suggest that a stable interaction between CycI and Ycf54 is required for unimpeded Pchlide biosynthesis. Crystal structures of the WT, A9G and R82A Ycf54 proteins were solved and analysed to investigate the structural effects of these mutations. A loss of the local hydrogen bonding network and a reversal in the surface charge surrounding residue R82 are probably responsible for the functional differences observed in the R82A mutation. We conclude that the Ycf54 protein must form a stable interaction with CycI to promote optimal Pchlide biosynthesis.

## Introduction

**Figure 1.**
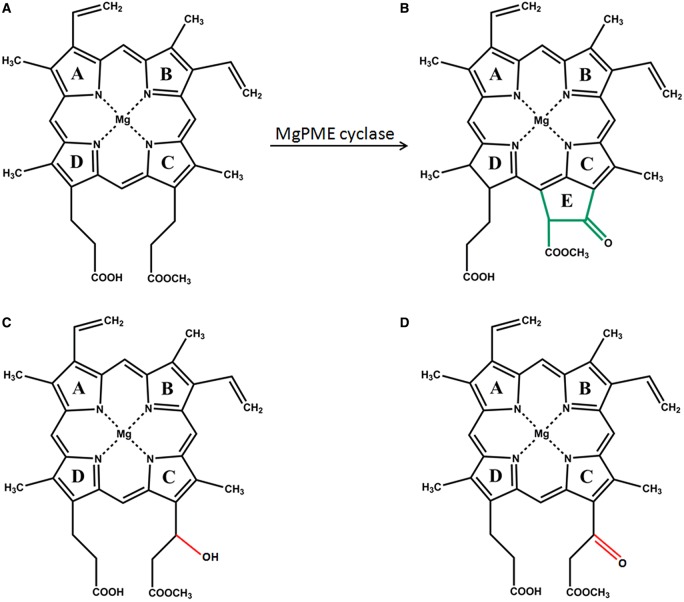
The MgPME cyclase reaction. The structures of the substrate, Mg-protoporphyrin IX monomethyl ester (**A**); product, protochlorophyllide (**B**) and proposed reaction intermediates of the oxidative cyclase reaction. In the model proposed by Granick (43), supported by the observations of Wong and Castelfranco (38), the conversion of MgPME to Pchlide proceeds through three sequential two-electron oxidations, sequentially passing through the intermediates Mg-protoporphyrin IX 6-methyl-β-hydroxypropionate (**C**) and Mg-protoporphyrin IX 6-methyl-β-ketopropionate (**D**).

Photosynthesis is dependent on chlorophylls (Chls), the most abundant light-absorbing pigments on Earth. All Chls are modified tetrapyrrole molecules distinguished by their centrally chelated magnesium ion and isocyclic E or fifth ring. In oxygenic-photosynthetic organisms, Chls, haems and bilins share the same biosynthesis pathway up to protoporphyrin IX. At this branch point, the action of one of the two chelatases, Mg-chelatase or ferrochelatase, determines whether protoporphyrin IX is directed towards the Chl or the haem biosynthesis pathways. Chl biosynthesis is initiated by insertion of a magnesium ion into protoporphyrin IX by Mg-chelatase generating Mg-protoporphyrin IX, which is converted by Mg-protoporphyrin methyltransferase (ChlM) to Mg-protoporphyrin IX monomethylester (MgPME). Mg-protoporphyrin IX monomethylester cyclase (MgPME–cyclase) catalyses the formation of the isocyclic E ring by cyclising the methyl propionate side-chain at C-13 to the C-15 bridge carbon between rings C and D, generating protochlorophyllide (Pchlide) (Figure 1). The light-activated enzyme Pchlide oxidoreductase (POR) reduces Pchlide to chlorophyllide (Chlide), to which a polyisoprene tail is attached by Chl-synthase (ChlG). This produces Chl *a*, so concluding the Chl biosynthesis pathway [1,2].

Several enzymes involved in the Chl biosynthesis pathway have been characterised in detail; however, the MgPME–cyclase remains a notable exception. Biochemical analyses of this enzyme have been limited to assays using extracts from cucumber chloroplasts [3–7], wheat etioplasts [8], barley plastids [9], *Chlamydomonas reinhardtii* and *Synechocystis* sp. PCC6803 (hereafter *Synechocystis*) [10]. Partial purification of the MgPME–cyclase complex from these extracts showed that the enzyme contains at least one membrane-bound and one soluble protein [3,9,10]. One of the subunits was identified when inactivation of the *acsF* (aerobic cyclisation system Fe-containing protein) gene in *Rubrivivax gelatinosus* resulted in MgPME accumulation under aerobic conditions [11]. Subsequently, *acsF* homologues were found in many oxygenic-photosynthetic organisms including *Chlamydomonas reinhardtii* (*Crd1* and *Cth1*) [12], *Arabidopsis thaliana* (*Chl27*) [13], Barley (*Xantha-I*), *Synechocystis* (*cycI*/*sll1214* and *cycII*/*sll1874*) [14,15] and *Rhodobacter sphaeroides* (*rsp_0294*) [16]. The *AcsF* gene product encodes a membrane-associated di-iron protein, which resembles proteins within the mono-oxygenase family [12,13].

Recently, the small monomeric protein Ycf54 was identified as a second candidate component of the MgPME–cyclase [17–19]. Ycf54 was found to interact with the *acsF* homologues *cycI* and *cycII* in *Synechocystis* during pulldown experiments and CHL27 in *Arabidopsis* in biomolecular fluorescence complementation assays. Disruption of the *ycf54* locus in *Synechocystis* (*slr1780*) [19] and *Arabidopsis* (*LCAA*) [17] led to accumulation of MgPME and reduced synthesis of Pchlide and Chl *a*, although the lower levels of the respective AcsF homologues arising from loss of Ycf54 could account for the observed decrease in MgPME–cyclase activity [17,19,20]. In *C. reinhardtii*, the *ycf54* homologue *cgl78* is up-regulated under copper-deficient conditions [18,21], although *ycf54* transcripts were not identified as differentially expressed under reduced or replete copper conditions in *Synechocystis* [22]. Thus, the role of Ycf54 is unclear and indeed photoheterotrophic bacteria that contain an AcsF-type cyclase such as *Rubrivivax gelatinosus* do not appear to contain a Ycf54 homologue, bringing into question the essential nature of Ycf54 as a component of the Mg-PME cyclase.

The present study investigates whether Ycf54 is an essential component of the MgPME–cyclase in oxygenic-photosynthetic organisms, or if this protein is required for stability of the AcsF subunit and consequently MgPME–cyclase activity. Seven highly conserved residues within the Ycf54 coding region were identified, systematically mutated *in vivo* and their effect on MgPME–cyclase activity determined. Deposited in the PDB are two structures for Ycf54 homologues from *Thermosynechococcus elongatus* (PDB 3HZE) and *Nostoc* sp. (PDB 3JSR). However, as no work has been performed relating the structure and function of Ycf54, we also solved the crystal structures of *Synechocystis* wild-type (WT) Ycf54 and two Ycf54 mutants (A9G and R82A), both of which displayed a phenotype *in vivo*. Together, the *Synechocystis* Ycf54 point mutants and a series of *in vivo* pull-down experiments using native and mutant Ycf54 proteins, show three of the seven conserved residues (D39, F40 and R82) are required for Ycf54 to interact with the AcsF subunit and for *Synechocystis* to produce Pchlide.

## Experimental

### Growth conditions

*Synechocystis* strains were grown aerobically in an illuminated shaker at 30°C, under normal (50 µmol photons m^−2^ s^−1^) or low light conditions (4 µmol photons m^−2^ s^−1^) in liquid BG-11 [23] media supplemented with 10 mM TES (pH 8.2) and 5 mM glucose.

*E. coli* BL21 p*Lys*S [24] strains were transformed with the pET14b plasmid grown in super Luria-Bertani media, supplemented with 50 µg ml^−1^ ampicillin and 34 µg ml^−1^ chloramphenicol (Cm). Recombinant proteins were produced by overexpression for 24 h at 28°C with rotary shaking; no induction was required.

### Cloning and site-directed mutagenesis

The *ycf54* gene was amplified by PCR, using the primers *ycf54* F and *ycf54* R, from *Synechocystis* genomic DNA and cloned into the pET14b plasmid so as to introduce an N-terminal His_6_-tag. The A9G and R82A point mutations were introduced using the Stratagene QuikChange® kit, with the primers: Ala9Gly F, Ala9Gly R, Arg82Ala F and Arg82Ala R.

Plasmid pPM-*ycf54* (Supplementary Figure S1) was constructed by amplifying the *ycf54* gene with the region 300 bp directly upstream (using primers *ycf54* US F and *ycf54* US R) and the region 500 bp directly downstream (using primers *ycf54* DS F and *ycf54* DS R) of *ycf54*). These two fragments were ligated into pET3a, so as to flank a chloramphenicol resistance cassette (amplified with primers *Cm^R^* F and *Cm^R^* R) inserted at the multiple cloning site. The point mutations A9G, F13A, E22A, E26A, D39A, F40A, R82A were inserted using the Stratagene QuikChange® kit, with the primers listed in Supplementary Table S2. The pPM-ycf54 plasmid was transformed into the Δ*ycf54* *Synechocystis* strain and transformants were selected on BG-11 agar plates containing 10 μg ml^−1^ Cm. Full segregation was achieved by selection on increasing concentrations of Cm to a final concentration of 160 μg ml^−1^ Cm.

The pPD-FLAG-Ycf54 plasmid was constructed as described in [19] and the A9G, D39A, F40A and R82A point mutations inserted using the Stratagene QuikChange® kit. The plasmids were transformed into the *Δycf54 Synechocystis* strain and transformants were selected on BG-11 agar plates containing 10 μg ml^−1^ neomycin. Full segregation was achieved as described above.

### Extraction of pigments and quantification of Chl *a*

Cultures standardised by OD_750_ were pelleted in mid-exponential phase and Chls were extracted from the cell pellets, after washing in distilled H_2_O, by adding 10 volumes of 0.2% ammonia in methanol, vortex-mixing for 30 s and incubating on ice for 20 min. The extracts were clarified by centrifugation (15 000×***g*** for 5 min at 4°C) and the supernatants were immediately analysed on an Agilent 1200 HPLC system. The Chl content was calculated from spectrophotometric data by the method of Porra et al. [25].

Chl precursor pigments were separated on a Phenomenex Aqua C18 reverse-phase column [5 μM particle size, 125 Å (1 Å = 0.1 nm) pore size, 250 mm × 4.6 mm], using a method modified from Sobotka et al. [26]. Solvents A and B were 350 mM ammonium acetate and 30% methanol (v/v) and 100% methanol, respectively. Pigments were eluted at 1 ml min^−1^ at 40°C on a linear gradient of 65–75% solvent A over 35 min, increasing to 100% to wash the column. Elution of Chl precursor species was monitored by checking absorbance at 416, 433 and 665 nm.

### Purification wild-type and mutant Ycf54 mutant proteins

Cell pellets from *E. coli* cultures overproducing Ycf54 proteins were re-suspended in binding buffer (50 mM Tris, pH 7.4, 500 mM NaCl, 10 mM imidazole) containing EDTA-free protease inhibitor tablets (Roche), and the cells disrupted on ice by sonication. The lysate was clarified by centrifugation at 40 000×***g*** for 30 min at 4°C. The soluble fraction was applied to Chelating Sepharose FastFlow resin (GE Healthcare) equilibrated with NiSO_4_. The column was washed first with binding buffer, then a 50 mM imidazole wash, followed by a 100 mM imidazole wash and eluted with 500 mM imidazole. The eluted proteins were buffer exchanged into PBS and the N-terminal His_6_-tag was removed by cleaving with Thrombin 80 U ml^−1^ (GE Healthcare) at room temperature overnight. The sample was re-applied and washed through the Chelating Sepharose resin and the cleaved protein was buffer exchanged into 100 mM NaHCO_3_, pH 8.3, 50 mM NaCl buffer and concentrated to 10 mg ml^−1^ for crystallisation.

FLAG pulldown experiments were performed on solubilised thylakoid membranes prepared from cells harvested from 8L cell culture as described in [19].

### 77 K low temperature fluorescence spectroscopy

*Synechocystis* cultures, pelleted in mid-exponential phase, were re-suspended in 80% glycerol (v/v) to an OD_750_ = 0.1. UV-VS fluorescence spectroscopy was performed in a SPEX Fluorolog spectrofluorometer (SPEX Industries Inc.) with a xenon light source. Cell suspensions were cooled to 77 K in an OptistatDN nitrogen bath cryostat (Oxford Instruments, Oxford, U.K.). Emission spectra were recorded from cells excited at 435 nm with 5 nm slit widths, scanning between 450 and 900 nm.

### Clear-native electrophoresis and immunodetection

Membrane and soluble protein fractions were isolated from 50 ml of cells at OD_750 nm _∼ 0.6 according to [27] using 25 mM sodium phosphate, pH 7.4, 50 mM NaCl, 10 mM MgCl_2_, 10% glycerol (w/v) supplemented with EDTA-free protease inhibitor (Roche). Isolated membrane complexes (6 mg ml^−1^) were solubilised by the addition of *n*-dodecyl-β-d-maltoside to a final concentration of 2% (v/v).

To assess protein levels by immunodetection, the protein content of *Synechocystis* lysates was quantified spectroscopically [28], separated by SDS-PAGE (Novagen) and transferred to a nitrocellulose membrane. The membranes were probed with specific primary antibodies and then with secondary antibodies conjugated to horseradish peroxidise (Sigma). The primary antibodies used in the present study were raised in rabbits as described in [19], with the exception of CHL27 (anti-CycI), which was purchased from Agrisera (Sweden).

Clear-native (CN) electrophoresis was performed essentially as described in [29]. Chl-binding proteins separated in the gel were visualised by excitation at 660 and 549 nm in a Gel Doc XR^+^ (BioRad) and analysed using the Image Lab™ software (BioRad).

### Structural biology

Purified Ycf54 and the mutant derivatives were screened for crystal formation employing the vapour diffusion method at a 1:1 ratio of protein solution to mother liquor. Crystals of WT Ycf54 grew in 0.2 M ammonium fluoride and 2.2 M ammonium sulphate. Crystals of A9G Ycf54 grew in 0.1 M trisodium citrate and 2.4 M ammonium sulphate, while crystals of R82A were produced from 2.2 M ammonium sulphate alone. Crystals were transferred to a cryoprotectant solution comprising mother liquor with the inclusion of 30% ethylene glycol. These were mounted for data collection at 100 K.

Data were collected at Diamond light source on beam line I04, for the WT and I02 for the A9G and R82A Ycf54 crystals. The data from the WT, A9G and R82A protein crystals were collected to resolutions of 1.3, 1.5 and 2.2 Å, respectively (Table 1). The data were integrated and scaled using the Xia2 programme [30] and the structures were determined by molecular replacement using Phaser [31] from the CCP4 package [32] with the Ycf54 structure from *Thermosynechococcus elongatus* (3HZE) as the search model. The WT and A9G Ycf54 crystals were in space group *C*222_1_, with one monomer in the asymmetric unit, whereas the R82A Ycf54 crystal was in space group *P*2_1_2_1_2_1_ and contained four monomers in the asymmetric unit. Refmac5 [33] and Coot [34] were used for rebuilding and refinement, with structure validation performed in Molprobity [35]. The final models for each structure contain all atoms for residues 1–106; the R factor and R free for each structure are 0.15, 0.18 (WT); 0.18, 0.24 (A9G) and 0.21, 0.25 (R82A) (Table 1). The three structures have been deposited in the PDB with accession codes 5M2P, 5M2R and 5M2U, for WT, A9G and R82A, respectively.

**Table 1.** Data processing and refinement statistics

Data collection	WT	A9G	R82A
Wavelength (Å)	0.9686	0.97949	0.97949
Resolution range (Å)^3^	59.6–1.33 (1.36–1.33)	24.5–1.50	27.8–2.2
Space group	*C*222_1_	*C*222_1_	*P*2_1_2_1_2_1_
Unit cell (a,b,c, Å; α,β,γ, °)	42.7, 45.9, 119.3; 90, 90, 90	42.2,46.1,119.7; 90,90,90	55.5, 91.2, 120.3; 90, 90, 90
Total reflections^3^	122 617 (5703)	114 130 (8496)	206 333 (15 471)
Unique reflections^3^	27 322 (1916)	18 771 (1330)	31 240 (2237)
Multiplicity^3^	4.5 (3.0)	6.1 (6.4)	6.6 (6.9)
Completeness (%)^3^	99.5 (95.6)	99.0 (98.2)	99.1 (98.5)
Mean I/σ (I)^3^	16.5 (2.2)	10.9 (2.3)	22.6 (2.8)
Wilson B factor	8.7	17.9	42.1
*R*_merge_^1,3^	0.05 (0.52)	0.08 (0.77)	0.05 (0.79)
*R*_pim_^2,3^	0.03 (0.38)	0.04 (0.35)	0.02 (0.35)
Refinement			
R_factor_	0.15	0.18	0.21
R_free_	0.17	0.24	0.25
No. of non-H atoms	1022	969	3593
Protein	895	880	3436
SO_4_	5	5	–
Water	122	84	157
Protein residues	106	106	425
RMSD (bonds) (Å)	0.01	0.011	0.012
RMSD (angles)(°)	1.42	1.38	1.47
Ramachandran favored/allowed (%)	96.3	97.2	97.6
Ramachandran outliers (%)	0	0	0
Molprobity score	1.24 (94th percentile N = 2319, 1.33 Å ±0.25 Å)	0.87 (100th percentile N = 4836, 1.50 Å ±0.25 Å)	1.16 (100th percentile N = 10167, 2.20 Å ±0.25 Å)
Average B factors (Å)^2^			
Main chain	11.0	20.7	48.2
Side chains	17.4	26.5	52.6
SO_4_	19.4	66.6	–
Water	26.3	32.1	52.0
PDB code	5M2P	5M2R	5M2U

1 *R*_merge_ = Σ*_hkl_* Σ*_i_*|*I_i_* − *I*_m_|/Σ*_hkl_*Σ_i_ *I*_i_.

2 *R*_pim_ = Σ*_hkl_*√1/*n*−1Σ*_i_*_=1_|*I*_i_ − *I*_m_|/Σ*_hkl_*Σ_i_ *I*_i_, where *I*_i_ and *I*_m_ are the observed intensity and mean intensity of related reflections, respectively.

3 Values in parenthesis are for data in the high-resolution shell.

## Results

### Site-directed modification of seven highly conserved residues in Ycf54

**Figure 2.**
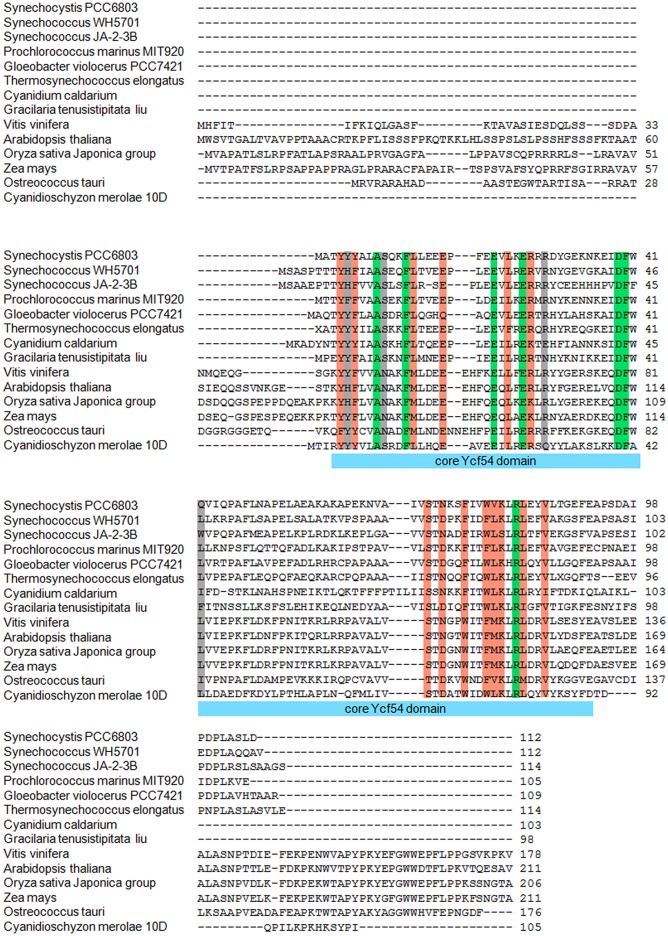
Amino acid sequence alignments (aligned using CLUSTALW2 [44]) of proteins predicted to contain the Ycf54 domain. Sequences were obtained from primordial cyanobacterium, *Gloeobacter violoceus*; cyanobacteria, *Synechocystis* sp. PCC6803, *Synechococcus* sp. WH5701 and JA-2-3B, *Prochlorococcus marinus* MIT920 and *Thermosynechococcus elongatus*; green plants, *Vitis vinifera*, *Arabidopsis thaliana*, *Oryza sativa* and *Zea mays*; red algae *Cyanidium caldarium*, *Cyanidioschyzon merolae* and *Gracilaria tenusistipitata* and the green alga *Ostreococcus tauri*. Conserved, highly similar and similar residues are highlighted in green, orange and grey, respectively.

Ycf54 homologues from a diverse range of photosynthetic organisms were identified via a BLAST search and aligned using ClustalW2, revealing a conserved core domain of 90 residues that contains 7 very highly conserved residues (Figure 2). With reference to the *Synechocystis* Ycf54 primary sequence, these conserved residues are A9, F13, E22, E26, D39, F40 and R82 (Figure 7).

To determine which of these residues play a role in Ycf54 function or are required for Pchlide formation, we generated a series of *Synechocystis* strains that express the native *ycf54* gene with a point mutation substituting each one of the seven conserved residues for an alanine, or in the case of A9, a glycine (Supplementary Figure S1). Full segregation of each of the *ycf54* point mutation strains was confirmed by PCR amplification of the *ycf54* region (Supplementary Figure S1), followed by sequencing of the amplified PCR product.

### *Ycf54* point mutants D39A, F40A and R82A have reduced Chl and accumulate MgPME, the substrate of the MgPME–cyclase

**Figure 3.**
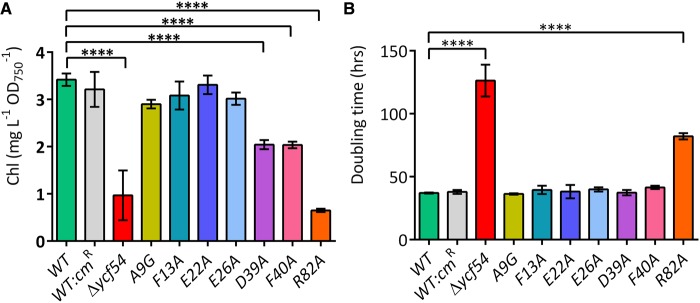
Chl *a* content and photosynthetic growth rates of the *ycf54* mutants. (**A**) The cellular Chl *a* level and (**B**) doubling time calculated for cells grown photoautotrophically in BG11 medium at a light intensity of 50 µmol photons m^−2^ s^−1^. Chl *a* was quantified using the method of [22] and doubling time was calculated from OD_750_ readings taken every 12 h for a total of 108 h. *P* values were calculated in GraphPad Prism version 6.00 using a one-way ANOVA followed by Dunnett's multiple comparisons test (****, *P* ≤ 0.0001).

To investigate whether any of the *ycf54* point mutations affect Chl accumulation or photoautotrophic growth, we ascertained the cellular Chl levels and doubling times for each strain under photoautotrophic growth conditions (Figure 3). We observed a significant reduction (*P* ≤ 0.0001) in the cellular Chl levels of mutants D39A, F40A and R82A, whilst the cellular Chl levels for mutants A9G, F13A, E22A and E26A were similar to the WT (Figure 3A). Of the mutants with reduced Chl, only R82A exhibited significantly reduced growth (*P* ≤ 0.0001) when compared to WT (Figure 3B). Although the cellular Chl content of mutants D39A and F40A was reduced by 40%, this has no significant effect on the photoautotrophic growth rate (Figure 3B). The 684 nm absorbance peak in the whole-cell absorbance spectra (Figure 3A) follows the same pattern as the cellular Chl contents shown in Figure 3A; R82A contains the least Chl of all the point mutants, which is reduced to the level of Chl in Δ*ycf54*.

**Figure 4.**
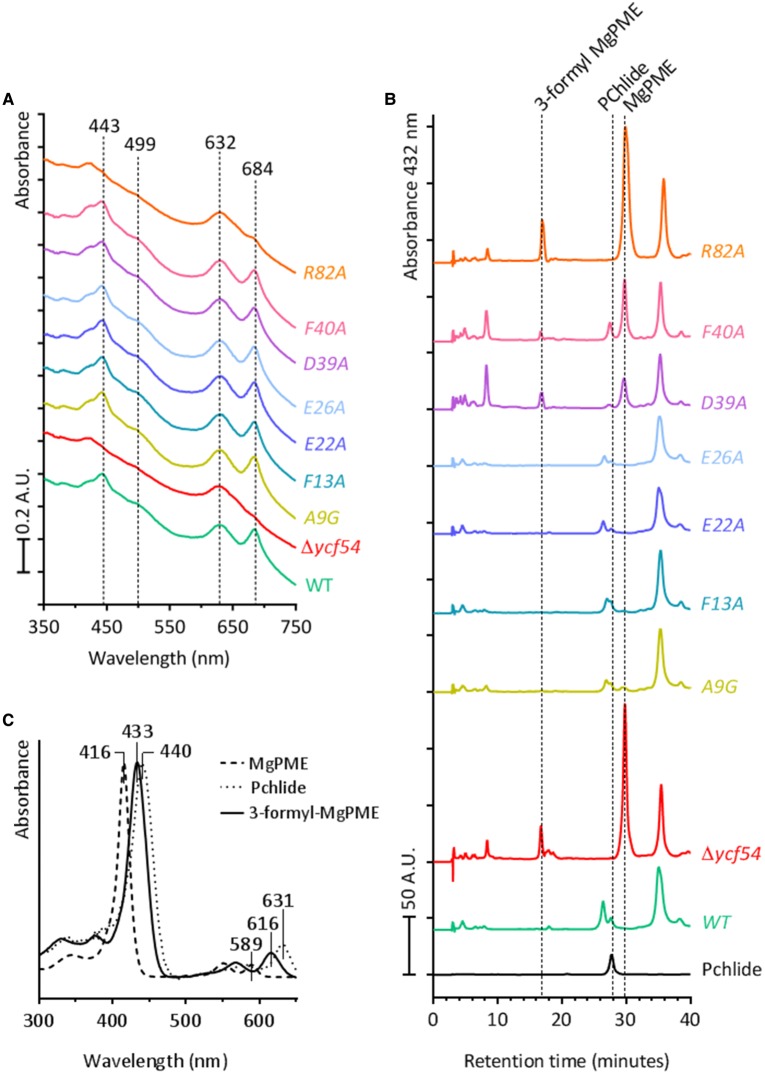
Absorbance spectra and HPLC analyses of pigments extracted from *ycf54* mutant strains. (**A**) Whole-cell absorbance spectra recorded for samples normalised to light scattering at 750 nm. (**B**) HPLC analyses of methanol extracted pigments from WT and ycf54 mutants grown under photomixotrophic conditions and normalised for absorbance at OD_750_. The retention times of peaks of interest are indicated. (**C**) Absorbance spectra of 3-formyl MgPME, MgPME and Pchlide, the absorbance maxima for each pigment are indicated.

Previously, we reported that a *Synechocystis* Δ*ycf54* mutant was disrupted at the step of the MgPME–cyclase in the Chl biosynthesis pathway. This fully segregated Δ*ycf54* mutant accumulated very high levels of MgPME, the substrate of the MgPME–cyclase and only trace amounts of Pchlide, the product of the MgPME–cyclase [20]. To ascertain whether any of the *ycf54* point mutants exhibited similar phenotypes, cellular pigments were methanol extracted from whole cells and analysed by HPLC (Figure 4B). HPLC analysis of extracts from mutants D39A, F40A and R82A showed distinct peaks at 16.5 and 29.8 min, which are analogous to the peaks in the Δ*ycf54* mutant extract (Figure 3B). The 416 nm absorbance maxima of the peak at 28.9 min is consistent with the observed absorbance maximum of MgPME (Figure 4C) and the 433 nm absorbance maxima of the peak at 16.5 min is consistent with 3-formyl-MgPME (Figure 4C), which was found to accumulate in the *Synechocystis* strain Δ*ycf54* [20]. Interestingly, the HPLC chromatograms of D39A and F40A both contain detectable levels of Pchlide, but this pigment was not detected in chromatograms of Δ*ycf54* and R82A. This suggests that the mutants are blocked at the MgPME–cyclase step with differing severity; mutants D39A and F40A exhibit a ‘leaky’ MgPME–cyclase phenotype and mutants R82A and Δ*ycf54* exhibit a severe MgPME–cyclase phenotype.

### *Ycf54* mutants Δ*ycf54*, A9G and R82A affect the accumulation of MgPME–cyclase components and the photosystems

**Figure 5.**
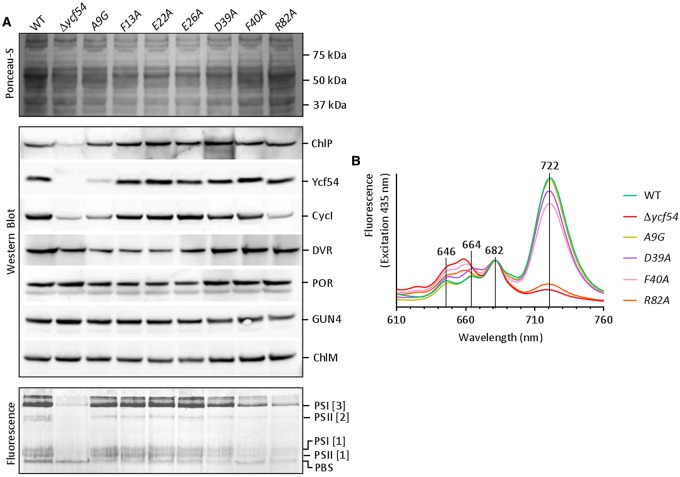
Accumulation of *Chl* biosynthesis enzymes and photosystem complexes in *ycf54* mutant strains. (**A**) Whole-cell lysates prepared from each of the *ycf54* mutant strains containing equal quantities of protein were separated by SDS-PAGE and transferred to a nitrocellulose membrane. The proteins ChlP, Ycf54, CycI, DVR, POR, GUN4 and ChlM were detected by specific antibodies. Membrane fractions isolated from each of the *ycf54* mutant strains were separated by CN-PAGE and visualised by recording fluorescence emission at 680 and 549 nm. Indicated are the PSI and PSII complexes. (**B**) 77 K whole-cell emission spectra from *Synechocystis* strains WT, Δ*ycf54*, A9G, D39A, F40A, R82A. Emission spectra were recorded for excitation at 435 and 580 nm. For comparability the 435 nm spectra were normalised to PSII emission at 682 nm and the 580 nm spectra were normalised to phycocyanin emission at 646 nm.

To investigate whether the blockage at the MgPME–cyclase step is a direct result of the point mutations in *ycf54* or is a result of a reduction in the known MgPME–cyclase component CycI, we probed western blots of the whole cell lysates with antibodies to Ycf54 and CycI, as well as the Chl biosynthesis enzymes GUN4, ChlM, POR, divinyl reductase (DVR) and geranylgeranyl reductase (ChlP) (Figure 5A). These blots show that the mutant Ycf54 proteins, with the exception of A9G, accumulate at levels similar to WT Ycf54. The reduction in Ycf54 to ∼25% of the WT level in mutant A9G is intriguing, as Ycf54 expression is not significantly perturbed in strain A9G (assessed by RT-PCR, Supplementary Figure S2) and an immunoblot of recombinant WT and A9G Ycf54 proteins shows that both are detected equally well by the Ycf54 antibody (Supplementary Figure S2).

The concurrent reduction of Ycf54 and CycI in strain A9G suggests that the presence of CycI is dependent on Ycf54. This ∼50% decrease of CycI in strain A9G is accompanied by a 15% reduction in cellular Chl when compared to WT (Figure 3A). The Δ*ycf54* and R82A mutants, which contain no Ycf54 and a point mutated Ycf54, respectively, also accumulate ∼50% less CycI than WT (Figure 5A). However, Chl accumulation in these strains is reduced by over 80%. It is unlikely that the lowered CycI levels in Δ*ycf54* and R82A could account for greatly diminished levels of Chl and photosystem complexes (Figures 3A and 5) given the impediment of cyclase activity in the F40A and D39A strains that accumulate CycI at levels comparable to WT. Together, these observations suggest Ycf54 has a dual role, its presence being required for both Pchlide formation and the stability of the catalytic cyclase subunit CycI.

In order to observe the effects of lowered Chl on photosystem accumulation, detergent-solubilised membranes from WT *Synechocystis* and each of the point mutants were separated by CN-PAGE (Figure 5A). Previously, significantly lower levels of PSI trimers and PSII monomers and dimers were found in Δ*ycf54* [20]. Figure 5A shows that assembly of PSI trimers is reduced with increasing severity in the Chl-deficient point mutants D39A, F40A and R82A, respectively, which is confirmed by the levels of PSI emission observed from each of these mutants at 722 nm in the low temperature fluorescence spectra (Figure 5B). The result of CN-PAGE analysis, shown in Figure 5A, also shows that F40A and R82A have lower levels of PSI/PSII monomers, although not to the extent observed in Δ*ycf54*.

### The Chl-deficient Ycf54 mutants are unable to interact with CycI, the catalytic subunit of the MgPME–cyclase

**Figure 6.**
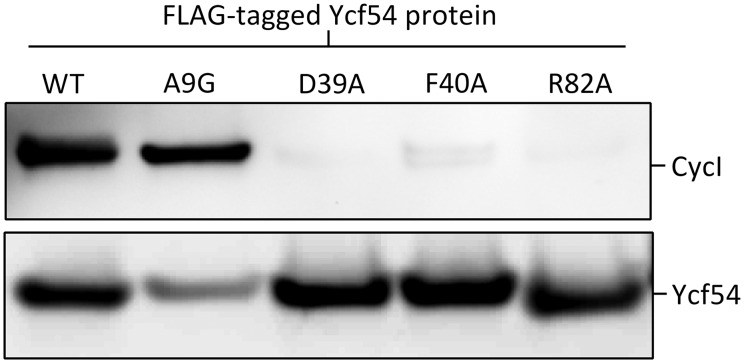
Ycf54 mutants D39A, F40A and R82A do not interact with CycI. FLAG-Ycf54 was purified from the dodecyl-β-maltoside solubilised membrane fractions from Synechocystis strains, FLAG-Ycf54, FLAG-Ycf54.A9G, FLAG-Ycf54.D39A, FLAG-Ycf54.F40A and FLAG-Ycf54.R82A. Eluted proteins from the FLAG-pulldown assays were resolved by SDS-PAGE and transferred by Western blot to a nitrocellulose membrane. The membrane was probed with anti-Chl27 (Agrisera), which detects CycI and anti-FLAG (Sigma), which detects FLAG-tagged Ycf54.

To ascertain whether MgPME–cyclase activity is dependent on the interaction between Ycf54 and the catalytic subunit of the MgPME–cyclase CycI, the Chl-deficient point mutants (D39A, F40A and R82A) and mutant A9G were 3x FLAG-tagged in the Δ*ycf54 Synechocystis* background. Pulldown experiments were performed on fractionated lysates from each of these strains and western blots were used to detect Ycf54 (using the FLAG antibody) and CycI (using the CHL27 antibody). Figure 6 shows that all the FLAG-Ycf54 mutant proteins, with the exception of A9G, accumulate at levels equivalent to WT FLAG-Ycf54, whereas FLAG-Ycf54.A9G, like its untagged counterpart (Figure 5A), accumulates at a reduced level. These pulldowns show that almost no detectable CycI is present in the pulldown experiments in which the FLAG-tagged Ycf54 mutants D39A, F40A and R82A were used as bait. Interestingly, although the FLAG-Ycf54.A9G protein is present in reduced quantities, this protein was found to pulldown a similar amount of CycI as WT FLAG-Ycf54. This suggests that MgPME–cyclase activity is dependent on the interaction of CycI with Ycf54, indicating that Ycf54 may either have a direct role in promoting the cyclase reaction or is required for the stability of the MgPME–cyclase complex.

### Structural characterisation of Ycf54

**Figure 7.**
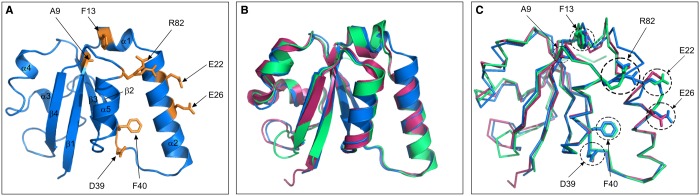
Structures of WT Ycf54 from *Synechocystis*, *Thermosynechococcus elongatus* and *Nostoc* sp. PCC 7120. (**A**) Cartoon of *Synechocystis* Ycf54 crystal structure, with the seven highly conserved residues highlighted in orange. (**B**) Superposition of the Ycf54 proteins from *Synechocystis* (blue), *Nostoc* (green) and *Thermosynechococcus elongatus* (pink). (**C**) Alignment of Cα backbone, showing the location of the seven highly conserved residues is conserved between species.

We crystallised the WT Ycf54 protein and solved the structure using molecular replacement to a resolution of 1.3 Å. *Synechocystis* Ycf54 is composed of a single domain (annotated as the Ycf54 domain in PFAM), in which a central four-stranded antiparallel β-sheet (β1–β4) is flanked on one side by helices α1, α2 and α5 and by helices α3 and α4 on the other (Figure 7A). This domain appears to be typical of the Ycf54 superfamily, with *Synechocystis* Ycf54 representing the most complete and highest resolution structure to date. Structural alignment of the *Synechocystis*, *Thermosynechococcus elongatus* BP-1 (PDB 3HZE) and *Nostoc* PCC 7120 (PDB 3JSR) Ycf54 proteins (Figure 6B) shows that they have a highly similar polypeptide fold with respective root mean square deviations (RMSD) for all Cα atoms when aligned with the *Synechocystis* WT Ycf54 structure of 0.68 Å and 0.66 Å. The conserved structural homology is further highlighted in Figure 7C, which shows that the seven residues of interest are located at highly conserved loci within the secondary structure.

An additional conserved structural feature is an electronegative ridge that extends across one face of the protein (Supplementary Figure S3). This ridge is located on α2 of *Synechocystis* Ycf54, where two of the highly conserved residues, E22 and E26, contribute to its negative charge. Although individually point mutating these residues to alanine yielded no discernible phenotype, the fact that the ridge is unusual and structurally conserved indicates that it may be of physiological importance. However, without further knowledge of how Ycf54 interacts with the other cyclase subunits, it is not possible to speculate on its function.

### A9G and R82A structures

A9G and R82 exhibited the most interesting phenotypes, in terms of near-normal Chl, lowered CycI levels plus retained interaction with CycI (A9G) or low Chl, lowered CycI levels and abolished interaction with CycI (R82). To investigate the structural basis for these effects of the A9G and R82A mutations in *Synechocystis,* crystal structures solved by molecular replacement against the WT *Synechocystis* Ycf54 were obtained for these two proteins to resolutions of 1.5 Å and 2.2 Å, respectively. Superposition of the A9G and R82A models on WT Ycf54 resulted in respective RMSDs of 0.19 Å and 0.28 Å over all Cα atoms, showing that there are no significant structural alterations between the WT and mutant structures. Thus, the phenotypes observed in A9G and R82A are not a result of the mutant proteins adopting a different conformation or failing to fold. Further analysis of the local structure around the A9G mutation shows that there are no alterations in the immediate hydrogen bonding network or surface electrostatics, indicating that the phenotypic consequences of the A9G mutation do not result from alterations in the structure.

**Figure 8.**
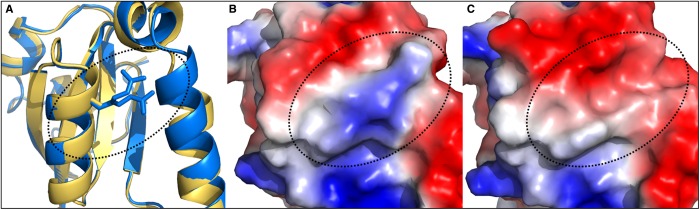
Ycf54 mutant R82A has altered surface charge. (**A**) Secondary structure superposition of the WT (blue) and R82A mutant (yellow) Ycf54 proteins. Indicated by the box is the R82A residue, which has a dual conformation in the WT structure. A comparison of the surface electrostatics surrounding the R82 region in WT Ycf54 (**B**), with the corresponding R82A region in R82A mutant Ycf54 (**C**), showing the change from positive (blue) to negative (red) surface charge in this area (black dotted oval, figure produced using Pymol [45]).

Upon closer inspection of the R82A structure, differences are observed in both the local hydrogen bonding network and surface electrostatics. In WT Ycf54, R82 adopts two clearly defined conformations (Figure 8A and Supplementary Figure S4A), both of which form stabilising hydrogen bonds with neighbouring residues. In one conformation, R82 forms a water-mediated hydrogen bond with the side chain of W78, and in the other R82 forms two direct hydrogen bonds with the backbone carbonyl of F20 and the side chain of E17 (Supplementary Figure S4A). All of these interactions are lost in R82A (Supplementary Figure S4B), which may alter the stability of this region. Examination of the surface electrostatics shows the base substitution to alanine changes the surface electrostatics from predominantly positive in WT Ycf54 (Figure 8B) to predominantly negative in R82A (Figure 8C). It may be that the flexibility of R82, along with its associated positive surface potential, is required for docking of Ycf54 onto CycI and consequently mediation of the cyclase reaction.

## Discussion

The Ycf54 protein has been identified as a component of the MgPME–cyclase complex. This protein was identified independently via its interaction with the AcsF homologue CycI [19] and in a screen for genes whose partial down-regulation resulted in a strong Chl deficiency in tobacco [17]. Although known to be an interaction partner of the AcsF component of MgPME–cyclase, it was not clear from these investigations whether Ycf54 was required for catalytic activity of the MgPME–cyclase, or if the protein was required to stabilise the MgPME–cyclase complex. Biochemical studies, using fractionated cell lysates, identified the MgPME–cyclase as consisting of a soluble component and at least two membrane components [3,7,9,10,36–38], of which the latter are likely to be AcsF and a protein encoded within the mysterious barley *viridis-k* locus [9]. Ycf54 is a candidate for the soluble protein, given its high solubility when expressed recombinantly*.* Conversely, *in vivo* studies reveal that this protein is localised in both the soluble and membrane fractions of *Synechocystis* cell lysate [20] and in barley the protein was found to form part of the membrane-bound component of the MgPME–cyclase [39], suggesting that the ‘true’ soluble component of the MgPME–cyclase remains to be found.

In this work, we generated a series of *Synechocystis* Ycf54 point mutants and solved the crystal structures of the WT protein and mutant proteins A9G and R82A to further elucidate the role of Ycf54 *in vivo*. Introduction of base substitution mutations that replace residues D39, F40 and R82 with alanine generated *Synechocystis* strains that were blocked at the MgPME–cyclase step to varying degrees (Figure 4B) and are deficient in Chl as a result (Figure 3A). A fourth Ycf54 mutant in which residue A9 was substituted with a glycine reduced Ycf54 levels by ∼75% and CycI levels by ∼50%. When FLAG-tagged and used as bait in pulldown experiments the three mutants with deficiencies in Chl biosynthesis, D39A, F40A and R82A, were unable to interact with the MgPME–cyclase component CycI. The FLAG-A9G construct, which like the A9G point mutation was present at lower cellular levels, pulled down CycI in quantities similar to those observed in the FLAG-Ycf54 control pulldown (Figure 6). These results explain to some extent the observable lack of a phenotype in A9G, as they suggest the level of Ycf54 forming a functional complex with CycI is consistent between FLAG-Ycf54 and FLAG-A9G. Indeed, previous pulldown experiments [19] showed that only FLAG-Ycf54 located within the insoluble fraction was capable of interacting with CycI and analyses of the sub-cellular localisation of Ycf54 showed that only a small minority of this protein was located in the insoluble membrane fraction [20]. Therefore, it is reasonable to conclude that although only a small amount of A9G accumulates, there is more than enough Ycf54 protein available to interact with CycI, so allowing the MgPME–cyclase to proceed unhindered.

We successfully crystallised WT Ycf54 and the two mutants A9G and R82A. Comparison of the WT and A9G structures found no structural reason for the low levels of A9G observed *in vivo*. As our pulldown experiments show A9G Ycf54 interacts with CycI at levels comparable to WT Ycf54, it is unlikely that the reduced level of A9G has a structural basis. One explanation for the lower level of A9G could be that the glycine codon inserted is rare in *Synechocystis*, resulting in lower levels of A9G Ycf54 translation. The structure of Ycf54.R82A, which has impaired Chl biosynthesis, ∼50% reduction in CycI levels and abolished interaction with CycI, reveals that the R82A mutation has reversed the normally positive surface electrostatics to yield an overall negative face. This alteration appears to prevent the docking of Ycf54 onto CycI, with consequences not only for the stability of CycI but also for turnover of the cyclase. The rest of the R82A Ycf54 structure remains unaltered by the arginine to alanine substitution; therefore, it is reasonable to conclude that this residue is required for Ycf54 to form a stable interaction with CycI. On the WT Ycf54 structure, residues D39A and F40A are not particularly surface exposed, so we are unable to speculate as to how these mutations result in a reduced interaction with CycI.

From this work, it appears that Ycf54 plays two roles in the function of the MgPME–cyclase. First, the accumulation of CycI is dependent on the presence of Ycf54, which suggests that Ycf54 may play a critical role in the assembly/stability of the Mg–cyclase complex and its constituents. Secondly, Ycf54 is required for normal Pchlide formation, indicating that this protein is required for optimal MgPME–cyclase activity, although it is not absolutely essential for catalysis. Earlier in the Chl biosynthesis pathway, the GUN4 protein promotes the activity of the Mg-chelatase, by lowering the Mg^2+^ threshold required for Mg-chelatase activity [40–42] and stimulating Mg-chelatase activity under physiological conditions. Thus, it could be the case that the role of Ycf54 in the MgPME–cyclase is analogous to GUN4 and Mg-chelatase.

## Abbreviations

3-Formyl-MgPME: 3-Formyl-magnesium-protoporphyrin IX monomethylester
Chl: chlorophyll
Chlide: chlorophyllide
ChlG: chlorophyll synthase
ChlM: Mg-protoporphyrin methyltransferase
Cm: chloramphenicol
DVR: divinylreductase
HPLC: high-performance liquid chromatography
MgPME: magnesium-protoporphyrin IX monomethylester
MgPME–cyclase: magnesium-protoporphyrin IX monomethylester (oxidative) cyclase
Pchlide: protochlorophyllide
POR: protochlorophyllide oxidoreductase
RT-PCR: reverse transcription polymerase chain reaction
*Synechocystis*: *Synechocystis* sp. PCC 6803
WT: wild type.
